# Spotting the differences: Probing host/microbiota interactions with a dedicated software tool for the analysis of faecal outputs in *Drosophila*

**DOI:** 10.1016/j.jinsphys.2014.05.023

**Published:** 2014-10

**Authors:** Matthew T. Wayland, Arnaud Defaye, Joao Rocha, Satish Arcot Jayaram, Julien Royet, Irene Miguel-Aliaga, François Leulier, Paola Cognigni

**Affiliations:** a Department of Zoology, University of Cambridge, Downing Street, Cambridge CB2 3EJ, UK; b Institut de Biologie du Développement de Marseille-Luminy, CNRS UMR 6216/Aix-Marseille Université, 13288 Marseille, France; c MRC LMB, Francis Crick Avenue, Cambridge Biomedical Campus, Cambridge, Cambridgeshire CB2 0QH, UK; d MRC Clinical Sciences Centre, Imperial College London, London W12 0NN, UK

**Keywords:** Drosophila, Intestine, Faeces, Software, Microbiota, Infection

## Abstract

The intestinal physiology of *Drosophila melanogaster* can be monitored in an integrative, non-invasive manner by analysing graphical features of the excreta produced by flies fed on a dye-supplemented diet. This assay has been used by various labs to explore gut function and its regulation. To facilitate its use, we present here a free, stand-alone dedicated software tool for the analysis of fly excreta. The Ultimate Reader of Dung (T.U.R.D.) is designed to offer a flexible environment for a wide range of experimental designs, with special attention to automation and high-throughput processing. This software detects the distinctive changes in acid-base and water balance previously reported to occur in response to dietary challenges and mating. We have used T.U.R.D. to test the contribution of the bacterial environment of the flies to various intestinal parameters including the established diet- and mating-triggered responses. To this end, we have analysed the faecal patterns of flies reared in germ-free conditions, upon re-association with controlled microbiota and subjected to food-borne or systemic, non-lethal bacterial infections. We find that the tested faecal outputs are unchanged in all these conditions, suggesting that the impact of the bacterial environment on the intestinal features highlighted by faecal deposit analysis is minimal.

## Highlights

• The Ultimate Reader of Dung is a dedicated tool for the analysis of faecal spot data.
• Effects of diet and mating on excreta do not rely on the presence of gut microbiota.
• Gut microbiota and infections have little impact on faecal water content and pH.

## Graphical abstract

**Figure:**
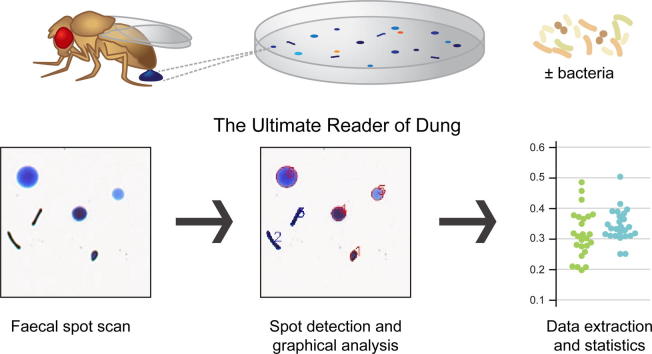

## 1 Introduction

In recent decades, the study of the genetic and mechanistic bases of energy homeostasis in invertebrate models such as insects has revealed deep molecular and functional conservation of nutritional responses, metabolic pathways and inter-organ signalling (Oldham, 2011, Rajan and Perrimon, 2013, Tennessen and Thummel, 2011). These findings have confirmed the relevance of these model systems to the study of physiology and metabolism, and have fuelled a blossoming of research aimed at exploiting the experimental assets of invertebrates to unravel the genetic and environmental regulation of homeostatic processes.

The fruit fly *Drosophila melanogaster*, combining a diminutive yet complex anatomy with simple maintenance and an unparalleled genetic toolset, is particularly suited for screening approaches aimed at bridging the gap between the genetic encoding and physiological manifestation of metabolic phenotypes. However, partly due to the small size that makes *Drosophila* such a practical model, most techniques traditionally used to assess metabolic state in larger organisms – such as the quantification of circulating metabolites or the direct measurement of ingested and excreted food – are not always practical in the fly, and often require the sacrifice of the animal. To circumvent this problem, we have previously developed a non-invasive method to monitor the intestinal physiology of *Drosophila* by analysing the faecal deposits produced by flies fed a diet supplemented with a dye (Cognigni et al., 2011). We used a pH-sensitive dye to highlight changes in acid-base balance in the hue of excreta. Changes in number, size, shape and dye concentration of the deposits are also reflective of changes in the flies’ intestinal biology, such as total excretion and water reabsorption (see (Cognigni et al., 2011) for a detailed description and validation of the biological correlates of faecal features). With appropriate controls, the faecal output can be used to assess food intake over a period of time in an undisturbed environment, offering a simple alternative to the more involved and often problematic techniques previously available (Wong et al., 2008). To analyse faecal features quantitatively, the excreta are imaged and processed digitally to identify individual deposits and extract their graphical features. A variety of commercial packages have been used for this process (Apger-McGlaughon and Wolfner, 2013, Cognigni et al., 2011, Slack et al., 2012, Urquhart-Cronish and Sokolowski, 2014), often depending on costly licenses and requiring extensive customisation of the analysis parameters to optimise them for faecal spot detection.

Faecal spot analysis has identified water retention as a novel hallmark of the female response to mating (Apger-McGlaughon and Wolfner, 2013, Cognigni et al., 2011) and has uncovered acidogenic effects of dietary challenges in the large intestine (Cognigni et al., 2011). Genetic analyses of these processes have shed light on some of the neuronal and genetic players involved in the regulation of intestinal function (Cognigni et al., 2011). These studies were performed in standard laboratory conditions which do not assess the role of – or changes in – the bacterial populations residing within the gut. In vertebrates, such bacteria are the most abundant members of the intestinal microbiota and have far-reaching roles in shaping intestinal physiology and the metabolic status of their host (Holmes et al., 2012). Recent work in *Drosophila* point to conserved roles in the fly (Lee et al., 2013).

In this study, we have developed a dedicated analysis tool based on open access libraries that we hope will facilitate the use of the faecal analysis technique for the *Drosophila* community. The Ultimate Reader of Dung (T.U.R.D.) automates the detection of the graphical parameters of faecal patterns and can be used to identify various established physiological changes reflected by alterations of the faecal pattern. We have then tested the effect of commensal and infectious bacteria by using T.U.R.D. to compare the faecal patterns of flies reared in axenic conditions or upon controlled re-association with standardized simple gut microbiota, and in the context of natural (food-borne) or systemic (injury-induced) non-lethal infections that are reported to activate a productive immune response.

## 2 Materials and methods

### 2.1 Image analysis

The Ultimate Reader of Dung is written in C++ using OpenCV, AlgLib (Bochkanov) and the Qt Framework (Qt project), and is released with a GNU General Public License. We encourage researchers to take advantage of its open source nature to develop it further and adapt it to their specific needs, under the terms of the GNU GPL. The software can be downloaded from http://sourceforge.net/projects/the-ultimate-reader-of-dung/, together with a collection of faecal pattern sample images. Refer to http://the-ultimate-reader-of-dung.sourceforge.net/# for installation and quick start instructions as well as additional documentation on software design and algorithm implementation. The Ultimate Reader of Dung is currently available for Windows and Mac OSX systems.

T.U.R.D. detects faecal spots by applying an adaptive threshold to the greyscale-transformed image. An adaptive rather than global threshold is used to compensate for local variation in background intensity. This thresholding results in a binary map of the image where background and foreground pixels are set to 0 and 1, respectively. Confluent regions of foreground pixels are labelled as faecal spots. Further refinement protocols are applied to the detected objects to reduce artefacts such as holes and confluent spots. The binary map is then used to extract features from the original image. Each object is analysed for shape, colour and position. The graphical variables extracted by the software, their definition and possible values, and their biological correlate when applicable, are listed in Table 1. Unless otherwise noted, refer to Cognigni et al. (2011) for validation of the biological relevance of the values.

**Table 1:** Graphical variables extracted by the software. Unless otherwise noted, refer to (Cognigni et al., 2011) for validation of the biological relevance of the values.

	Definition	Value/range	Biological correlate
Plate ID	A unique number associated with the plate image		
Deposit ID	A unique number associated with the object (see annotated image Fig. 1)	1–*n*	
*X* position	Position of the object in the image	Number of pixels from the top left corner	
*Y* position			
Area	Area of the object	Number of pixels	Size
Perimeter	Perimeter of the object	Contour approximation using the arcLength function (OpenCV, http://opencv.org/)	
Circularity	Similarity of the object to a perfect circle	(4π(area/perimeter^2^)) 0 (completely non-circular) to 1 (perfect circle)	Shape
ROD	Reproductive oblong deposit flag	0 (no) or 1 (yes) based on applying a user-defined cutoff to the Circularity value	Activation of the postmating water retention response (Cognigni et al., 2011)
IOD	Integrated Optical Density	(area * (1 − MeanL))	Total dye content of the deposit (Apger-McGlaughon and Wolfner, 2013)
MeanB	Mean colour of the object in the RGB (red–green–blue) model	0–1	
MeanG		0–1	
MeanR		0–1	
MeanH	Mean colour of the object in the HSL (hue–saturation–Lightness) model transformed from RGB according to (Agoston, 2005)	0–359	pH
MeanL		0 (black)–1 (white)	Dye concentration/diuresis
MeanS		0 (grey)–1 (fully saturated)	

Features that are not faecal spots can be present in the scan, such as small specks of dirt or dust or the shadows cast by the moulding of the plastic surface. Many of these can be automatically filtered out on the basis of their size: values for upper and lower size filtering can be specified by the user. Manual annotation of the sample plates reveals that, depending on the cleanliness of the plate, 10–15% (but occasionally up to 20%) of identified objects could be artefacts. These can be manually removed from the analysis by unchecking the “Include” checkbox of the object within the Plate Inspector window. The default filtering values have been optimised for the analysis of wild-type defecation patterns acquired at 1200 dpi; however, it may be necessary to adjust these in the case of manipulations that result in abnormally small or large deposits, such as alterations of fluid balance systems (Cognigni et al., 2011). In principle, the software can analyse images acquired at resolutions other than 1200 dpi; however, detection of deposits and especially shape features will be affected, and as such comparisons should always be drawn only between identically processed samples.

After identifying and analysing individual spots, T.U.R.D. compiles a collection of summary values for each image file Total values (normalised by the number of flies entered by the user) are calculated for number of faecal spots, area and IOD. Mean values for area, perimeter and shape features represent the arithmetic mean of the individual spot values. Mean colour information (in the RGB and HSL models) is obtained by analysing all spot pixels together and extracting the colour features of the resulting single object. While all these variables are computed during the plate-level analysis, only those values that have a direct biological correlate (see Table 1) are displayed in the basic output of the software. The full output, including additional graphical variables and the separate analysis of ROD and nonROD subsets, is also available and is always included in exported files.

### 2.2 *Drosophila* stocks and husbandry

*Drosophila* stocks were maintained in incubators at 25 °C, with 12 h/12 h light/dark cycles, on standard fly food (8.16 g/L agar, 80 g/L cornmeal, 80 g/L yeast extract, 5.2 g/L Nipagin, 4 mL/L 99% propionic acid). All experiments were carried out on adult flies of the *yw* double mutant stock, aged 7–9 days from eclosion. Males and females were separated at eclosion except for those marked as mated, which were kept in the presence of members of the opposite sex until the time of the experiment, i.e. 7–9 days.

The composition of specific diets used for this paper are the same as those described in Cognigni et al. (2011): 36 g/L yeast extract, 54 g/L sucrose for the Rich diet, 6 g/L yeast extract, 9 g/L sucrose for the Poor diet, and 90 g/L sucrose for the Sugar-only diet. 1.8 g/L Nipagin and 5 g/L Bromophenol blue sodium salt (Sigma, B5525) were added to all diets and pH was adjusted to 5.5.

### 2.3 Preparation of materials for faecal spot analysis

The excreta of adult flies are small, almost transparent droplets or semi-solid deposits. To aid their visualisation, the inexpensive, non-toxic dye Bromophenol blue sodium salt was added to the flies’ diet to a concentration of 5 g/L (Pulver et al., 2011). At this concentration, the dye does not affect the palatability of the food tested by two-choice CAFÉ assay and no developmental delay is observed if fed during the larval stage (data not shown). The dye is not altered by heat and can be added to liquid and solid diets. Its hue is however sensitive to pH, shifting from blue at neutral and basic pH to yellow and orange at acidic pH. Because of this, care should be taken in adjusting the pH of the prepared food to equal values across different conditions studied.

To obtain a digital image of fly excreta, groups of 6–8 flies were allowed to excrete onto flat clear plastic surfaces, in this case the lid Petri dishes, for about 60 h. The dye-laced food was offered ad libitum as a wedge of solid food placed on the dish (Pulver et al., 2011). At the end of the experiment, the flies and food were removed from the plate and a high-resolution image of the deposits left on the plastic surface was acquired with a transparency scanner (Epson Perfection V200). Image cropping and preparation were performed in Adobe Photoshop CS4.

### 2.4 Bacterial strains and cultures

In this study, the following bacterial strains were used: *Lactobacillus plantarum*^WJL^, *Lactobacillus brevis*^EW^, *Acetobacter pomorum*, *Commensalibacter intestini*^A911T^, *Erwinia carotovora carotovora*^15^-GFP, *Micrococcus luteus* and *Escherichia coli*^1106^ (Basset et al., 2000, Lemaitre et al., 1997, Ryu et al., 2008).

Lactobacilli were cultured in 40 mL of liquid MRS medium (Difco 288110) in a 50 mL tube (Falcon), at 37 °C, without agitation. *C.* *intestini*^A911T^ and *A.* *pomorum* were cultured in 50 mL of liquid Mannitol medium, (3 g/L Bacto peptone (Difco, 0118-17), 5 g/L yeast extract (Difco, 212750), 25 g/L D-Mannitol (Sigma, M1902), 15 g/L agar (optional)) in 250 mL or larger flasks at 29 °C, under 220 rpm agitation. *E.* *carotovora carotovora*^15^ and *Micrococcus luteus* were cultured in 200 mL of liquid LB medium (Difco 244610) in 500 mL or larger flasks at 29 °C, under 220 rpm agitation, using 10 g/L spectinomycin in the case of *E.* *carotovora carotovora*^15^-GFP. *E.* *coli*^1106^ was cultured in 200 mL LB at 37 °C with 220 rpm agitation. Cultures on plates were done on agar plates using the same culture medium and at the same temperature as for the liquid culture for each bacterium.

### 2.5 Generation of germ-free flies

Axenic flies were obtained by sterilizing embryos (2′ in 2.7% bleach, 2′ in 70% ethanol, 2′ in autoclaved distilled water) and placing them in vials containing standard food supplemented with a cocktail of antibiotics (final concentration: 50 μg/mL ampicillin, 50 μg/mL kanamicin, 10 μg/mL erythromycin, 50 μg/mL tetracycline). Antibiotics were added to the food while still liquid, but at a temperature below 60 °C. Established stocks of axenic flies were regularly transferred into vials containing freshly prepared antibiotic-supplemented food. The absence of detectable microbes in axenic stocks was regularly checked by grinding 10–15 flies in 500 mL of sterile LB culture medium, and spreading 100–200 μL of the homogenates on LB or BHI (Difco, 2372200) culture plates. After 48 h of incubation at 37 °C, the presence of bacterial colonies on the surface of the plates was checked.

### 2.6 Re-association of germ-free flies

Axenic flies were collected within the first 48 h of adult emergence and placed in groups of 25–30 females and 10 males on standard culture media without propionic acid. Either 150 μL of bacterial solution made of 75 μL of 5% sucrose sterilized by filtration through a 0.2 μm membrane (Pall Life Sciences, 4652) + 75 μL of a mix of 4 bacterial cultures (*L.* *plantarum*^WJL^, *L.* *brevis*^EW^, *C.* *intestini*^A911T^, and *A.* *pomorum*) at an initial optical density of 1.0 each, or 150 μL of a control solution (75 μL filter-sterilised sucrose + 75 μL of a 1:1 mixture of sterile MRS and sterile Mannitol) were added to a piece of autoclaved paper (Whatman 3030 917) and the treated paper was placed on top of the food. Flies were kept in such conditions for 7 days at 25 °C, and transferred into newly prepared vials with fresh bacterial cultures every 2 days (on day 3 and day 5). On day 5, the internal bacterial load was measured to check for the efficiency of the re-association or the axenic status of the flies.

### 2.7 Induction of food-borne and systemic infections

For food-borne intestinal infection, flies treated for 6 days according to the re-association protocol were starved in empty vials for 2 h at 29 °C. Half of the flies were subjected to food-borne infection by *E.* *carotovora carotovora*^15^ (Ecc15) by offering them a piece of filter paper containing 150 μL of a 1:1 mixture of Ecc15 (at an optical density of 200) and 5% sucrose; the remaining half received a control mixture of 1:1 LB and 5% sucrose as control. The flies were kept in these tubes at 25 °C for 24 h. After the faecal output experiment, 5 flies out of each set of 8 were ground to measure internal bacterial loads (commensals and Ecc15 loads).

Systemic infections were induced immediately before placing the flies into the faecal analysis Petri dishes by delivering a septic injury. Septic injuries were performed by pricking 7–9 days old conventionally reared flies in the lateral thoracic region with a thin needle previously dipped into a concentrated pellet of a 1:1 mixture of *M.* *luteus* and *E.* *coli*^1106^ at an optical density of 100 (50 final for each bacterium). Flies were then kept at 25 °C.

### 2.8 Measure of internal bacterial load

Internal bacterial loads were measured by spreading serial dilutions of fly homogenates on bacterial culture plates. Flies were first surface-sterilized by bathing them in 70% ethanol for 30 s under strong agitation. Flies were ground in groups of 5 in 500 μL of sterile bacterial culture medium (a 1:1 mixture of MRS and Mannitol medium) using the tissue homogenizer Precellys 24 (Bertin Technologies) and 100 μL of 0.75–1 mm glass beads, using two 20 s bouts at 6300 rpm separated by a pause of 25 s. The volume equivalent to 1 fly (100 μL) of the 1:1000 and 1:10.000 dilutions were spread over Mannitol plates (for *A.* *pomorum* and *C.* *intestini*^A911T^), another 100 μL on MRS plates (for Lactobacilli), and another 100 μL on LB plates supplemented with 10 μg/L spectinomycin in food-borne infection experiments (for the spectinomycin-resistant strain of *E.* *carotovora carotovora*^15^-GFP). Plates were incubated for 48 h at 37 °C (MRS) or 29 °C (Mannitol, LB-spec). Colonies formed over the plates were counted, and the internal bacterial load expressed as colony-forming units per fly. Bacterial loads of axenic flies were tested both before starting and after finishing the experiment.

## 3 Results and discussion

### 3.1 Analysing faecal spot data with The Ultimate Reader of Dung

Flies fed a diet supplemented with a dye will deposit their excreta as small spots that can be detected on a clear plastic surface (Cognigni et al., 2011). We sought to replicate and combine the graphical and statistical functions required for the extraction and analysis of faecal spot data without the use of commercial software. To do so, we have taken advantage of open source libraries to construct a stand-alone software tool referred to as The Ultimate Reader of Dung (T.U.R.D). T.U.R.D identifies spot objects from an image obtained from a scanner, extracts the graphical features of each individual spot and produces aggregate descriptions and statistical analysis of samples based on user-defined experimental groups. More details about the algorithms used for each of these steps are available on http://the-ultimate-reader-of-dung.sourceforge.net/#.

The presence of the pH-sensitive dye (Bromophenol blue) can be used to reveal changes in acid-base balance as shifts in the hue of the resulting deposits; likewise, the dilution of the dye will affect the colour intensity of the spots, offering a readout of water balance and fluid excretion (Cognigni et al., 2011). Furthermore, the amount of dye excreted by flies is necessarily a function of the amount of dye ingested. As long as there is no un-dyed food in the digestive system at the start of the experiment and internal gut capacity (the amount of dye retained within the digestive tract) is not changed, there is a direct relationship between food intake and dye excretion. Thus, with appropriate controls, food intake can be estimated from faecal output (Cognigni et al., 2011). To address the range of size and dye concentrations observed in individual faecal spot, it is possible to calculate an Integrated Optical Density (IOD) (Apger-McGlaughon and Wolfner, 2013) by multiplying the area of the faecal spot by its dye intensity (1 – mean Lightness). This calculation is only correct if the scanner settings and image preparation have been calibrated to ensure that the Lightness value is indeed a linear representation of dye concentration.

Previous work has identified a characteristic change in shape associated with reduced water content (Cognigni et al., 2011). The appearance of long, highly concentrated deposits is a hallmark of the female response to mating (Apger-McGlaughon and Wolfner, 2013, Cognigni et al., 2011) and these Reproductive Oblong Deposits or RODs can be identified by monitoring the shape of identified objects. The software will calculate a Circularity value to represent numerically the deposit shape and attribute each deposit to the ROD or non-ROD category on the basis of a user-defined cut-off. It should be noted that, while most deposits are unequivocally either round or elongated (such that the Circularity value typically displays a bimodal distribution), some excreta can be considered “transitional” (less elongated than indisputable RODs, but not perfectly round) and their manual classification is uncertain. In these cases, the use of a consistent numerical cut-off helps eliminate operator bias. The default cut-off value suggested by the software has been chosen empirically as the one that offer the best separation in our conditions: manual annotation indicates that, in experiments where ROD classification applies (i.e. those performed in females), >95% of excreta are correctly classified.

**Fig. 1:**
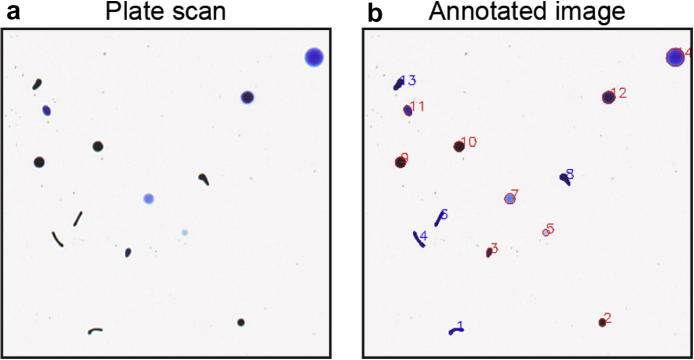
Image annotation with T.U.R.D. The analysis of the input image (a) identifies faecal spot objects which are outlined and labelled with their unique identifier on the annotated image generated by the software (b). RODs, as defined by their shape through a user-defined cut-off of the Circularity values, are colour coded by blue numbers.

After the analysis, the software generates an annotated image displaying each identified object, labelled with its unique Deposit ID and colour-coded with its ROD classification. An example of spot detection and annotation is shown in Fig. 1. The user can use such images to detect wrongly identified spots and remove them from further analysis.

### 3.2 Working with data sets

The faecal spot data extracted from each image file are processed to generate a summary of plate-level features. This associates the filename, ID, timestamp and detection protocol used for each processed image to the information extracted about biologically meaningful variables (namely number of spots, area, IOD, shape and colour parameters), calculated for the entire set of spots and for the ROD and non-ROD subsets separately.

The plate-level output of T.U.R.D. can be used to compare faecal patterns across plates and determine the effect of treatments or manipulations. To do so, each plate can be included in user-defined comparison groups. The software can calculate Descriptive Statistics for each group, displaying mean, standard deviation, median, minimum and maximum for each plate-level variable. Furthermore, differences between pairs of groups can also be highlighted with the Group Comparison option, which allows the user to select specific plate-level variables for contrast, using either parametric (Student’s *t*-test) or non-parametric (Mann–Whitney U test) statistics. To guide the choice of test, T.U.R.D. runs the Jarque–Bera normality test from the AlgLib library (Bochkanov), although care should be taken in inferring normality or lack thereof when working with small data sets.

To control for the familywise error rate associated with running multiple comparisons, the *p*-values calculated for each comparison are adjusted using the Holm–Bonferroni correction. Because this correction can be overly conservative and lead to Type II errors, it is useful to consider which variables are most relevant to the biological question at hand and restrict the analysis to those. This is particularly crucial when trying to show a lack of effect (see below), in which case the correction should not be applied.

**Fig. 2:**
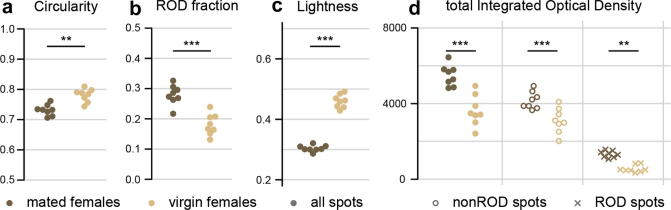
Mating-induced changes in faecal output analysed by T.U.R.D. Graphical features of faecal spots produced by mated (brown) and virgin (yellow) adult females. Each of the 8 replicates represents the faecal pattern produced by a set of 8 adult females. The spots produced by virgin females are more circular than those of mated females (a). This is due to the presence of Reproductive Oblong Deposits (RODs); individual RODs are automatically identified by T.U.R.D. on the basis of their Circularity value, and are significantly more frequent in mated female faecal patterns than in virgin females (b). A feature of the postmating response is water reabsorption (resulting in more concentrated, darker spots); this is reflected in a lower mean Lightness values in the faecal output of mated females (c). The total amount of excreted dye (Integrated Optical Density), an indirect measure of ingestion, is higher in mated females (d), consistently with the hyperphagic response triggered by mating signals. The increase involves both ROD and nonROD faecal spots. All comparisons are Student’s *t*-tests. All statistics (*t*-tests) were obtained with the T.U.R.D. group comparison function.

More advanced statistical treatment, especially important in the case of large datasets and multivariate experimental designs, should be carried out using dedicated statistical software. Each level of analysis in T.U.R.D. (deposit data, plate-level summaries, and descriptive and comparative statistics) can be exported in Comma Separated Value format and easily imported into various data analysis software packages. The exported files can also be used to generate graphical reports when entered into appropriate software. For example, Fig. 2 shows graphs generated in R (R Development Core Team, 2008) with faecal pattern information imported from the T.U.R.D. plate-level output.

### 3.3 High throughput processing

**Fig. 3:**
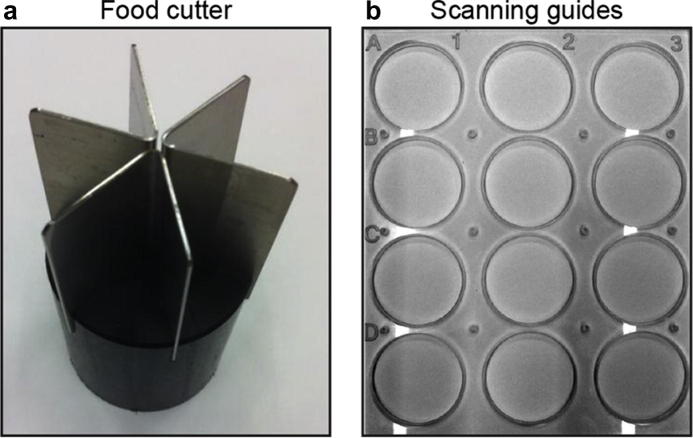
High-throughput resources. Food preparation can be standardised with a wedge cutter designed to fit a Petri dish of the desired size (a). A mould designed to house the Petri dishes can be used to standardise the position of plates on the scanner bed (b), which allows image cropping and preparation to be automated with image processing software such as ImageJ.

T.U.R.D. allows the user to process multiple plate images in batch and automatically generate annotated images and faecal spot reports, while keeping a record of the image files and parameters used for image processing to provide an audit trail. The data are organised by experiment in a relational database in order to deal with their hierarchical nature. The multi-thread architecture allows for parallel processing of images. We hope these advancements over the previous commercial software options will prove useful for higher-throughput designs such as screens. Other steps of the protocol, namely the preparation and scanning of the faecal spot plate, can also be optimised to facilitate high-throughput workflows. The assembly of the Petri dish setup can be simplified with the use of a food cutter (Fig. 3a) and image acquisition can be standardised with matching scanner guide moulds and digital masks (Fig. 3b and http://the-ultimate-reader-of-dung.sourceforge.net/#). With the use of macro-enabled image processing software such as ImageJ (Abramoff et al., 2004), these steps can be automated to extract and label multiple data files for each scan, ready to be entered into T.U.R.D. for batch processing. Further optimisations, such as labelling of plates with barcodes or QR codes, are available through open access resources and can be introduced into the image processing workflow.

### 3.4 Effect of gut microbiota on diet- and mating-induced changes in excreta

*Drosophila* naturally lives in micro-organism-rich ecological niches and carries a well characterised collection of commensal bacteria in its gut (Erkosar et al., 2013). Various reports indicate that, similarly to vertebrates (Holmes et al., 2012), the gut microbiota and food-borne bacterial infections are important elements impacting on gut homeostasis also in this organism (Buchon et al., 2009a, Lee et al., 2013), and could therefore alter the features of faecal outputs. Furthermore, as different subsets of micro-organisms will thrive on different diets and, consequently, different gut contents (Erkosar et al., 2013), it is possible that changes in commensal bacterial populations contribute to the alterations in faecal output previously associated with dietary manipulations (Cognigni et al., 2011). Likewise, the host response to an infection is affected by mating (Short and Lazzaro, 2013) so changes in immune function or bacterial loads could also contribute to the distinct faecal output observed in mated females (Fig. 2) and indicative of fluid retention (Cognigni et al., 2011).

**Fig. 4:**
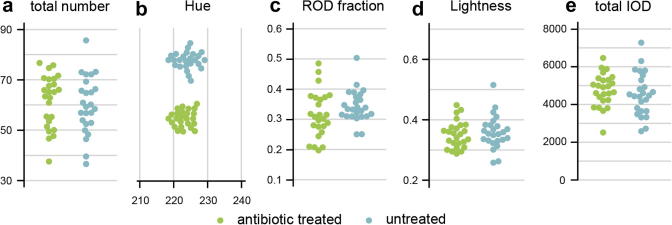
Impact of antibiotic feeding on faecal patterns. Feeding flies with a cocktail of antibiotics commonly used to eradicate the natural gut microbiota does not affect faecal output patterns. The number (a), colour (mean hue, b), ROD frequency (c), dye concentration (mean Lightness, d) and total amount of dye (total IOD, e) of treated flies (green) are undistinguishable from those of untreated flies (blue). Each of the 25 samples represents 8 mated adult females. All displayed variables (as well as mean Area and mean Circularity, not shown) were tested with the Student’s *t*-test provided by T.U.R.D.’s comparison functions (uncorrected to avoid Type II errors).

**Fig. 5:**
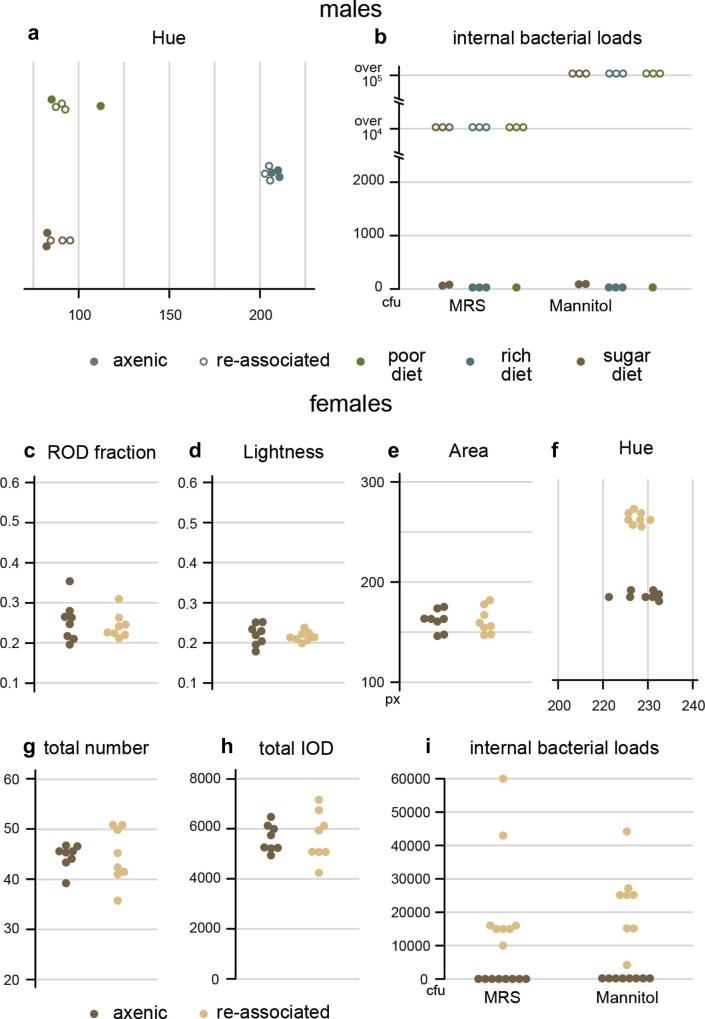
Diet- and mating-induced faecal output changes in germ-free and re-colonised flies. The faecal output of male flies is affected by dietary challenges, but not by gut microbiota status. Poor (green) and sugar-only (brown) diets result in acidification of excreta compared to the rich diet (blue), revealed by a shift of the mean hue variable towards lower (orange) values (a). The change in hue can be attributed almost entirely to the impact of diet, which accounts for 98.86% of the variance in a nested mixed linear model (with 0.14% of the variance being due to the presence of bacteria and 1% to individual variation not explained by either factor). Multivariate ANOVA performed in R reveals no significant effect of diet * microbiota interaction, indicating that germ-free (full circles) flies react to dietary changes in a manner indistinguishable from re-associated (empty circles) flies; however, the low statistical power of this interaction could be preventing the detection of effects. While the analysis reveals a significant effect of both Diet and microbiota separately, none of the appropriate pairwise comparisons (by Student’s *t*-test or Tukey’s Honest Significance test) shows a significant effect of microbiota in any of the diets tested. Each of the replicates represents the faecal pattern produced by a set of 6 adult males. Internal bacterial loads were tested after the experiment on MRS and Mannitol plates (b). Mated female flies produce their distinctive RODs independently of gut microbiota status. RODs are present in the same proportion (c) in the faecal output of axenic (brown) and re-associated (yellow) females and the concentration of spots (indicative of the postmating water reabsorption response) is also undistinguishable between the two groups as a whole (d) and for the ROD and non-ROD subsets (not shown). Faecal spots are equal in size (e), colour (f) and number (g) and the total dye excreted (h) is also identical, suggesting no effect of gut microbiota on food intake. All displayed variables (as well as mean Circularity, not shown) were tested with the Student’s t-test provided by T.U.R.D.’s comparison functions (uncorrected to avoid Type II errors). Internal bacterial loads were tested after the experiment on MRS and Mannitol plates (i).

To test the role of gut microbiota in the changes associated with different diets, we generated axenic flies by sterilising embryos with bleach and raising them on food supplemented with a mixture of antibiotics. This treatment does not, in itself, affect the faecal pattern of the animals raised on such antibiotics-supplemented food and further fed with it for one week after emergence (Fig. 4). These experiments were carried out in adult females, as this is standard practice in the study of *Drosophila* immunity and allows the detection of RODs, which are typical of mated females. When tested for biologically relevant outputs of T.U.R.D. analysis (refer to Table 1), no significant difference was revealed between treated and untreated flies. In parallel, we reconstituted a standardized gut microbiota by feeding germ-free animals with a mixture of four commensal strains isolated from lab-reared *Drosophila* (*L.* *plantarum*^WJL^, *L.* *brevis*^EW^, *C.* *intestini*^A911T^ and *A.* *pomorum*) (Ryu et al., 2008). The reconstitution was carried out after adult eclosion so as to avoid confounding developmental effects, and was confirmed by quantifying internal bacterial loads after each experiment (Fig. 5b and i). Adult flies were then exposed to three diets previously shown to result in distinctive faecal output changes (Cognigni et al., 2011): rich, poor (diluted to one sixth of the rich diet) and unbalanced (containing only sucrose). Male flies were used in this case for consistency with the published experiment. In challenging conditions (poor and sucrose diets), the blue colour of the pH-sensitive dye added to the food shifts to lower (orange–yellow) hue values, indicative of acidification of gut contents. By contrast, the faecal spots of flies fed rich food remain blue (neutral). Both germ-free and re-associated flies displayed the changes in the colour of excreta previously observed in conventionally reared flies fed a poor or sucrose diet (Fig. 5a). The changes in acid-base balance associated with poor and unbalanced diets are therefore not due to an interaction of these diets with gut microbiota. Likewise, mated germ-free female flies or females with reconstituted gut microbiota were still able to produce the typical Reproductive Oblong Deposits (RODs), and did so at identical levels (Fig. 5c), which were comparable to those observed in conventionally reared flies (compare Fig. 5 to Fig. 2). Other parameters of faecal output were also unaffected (Fig. 5d–h and not shown).

These results indicate that neither gut microbiota activity nor the host response triggered by their presence are responsible for the changes in excreta previously described in conventionally reared flies. However, our findings do not exclude bacterial effects on other aspects of intestinal physiology. Indeed, recent results suggest that gut microbiota impact digestive enzyme gene expression in the adult *Drosophila* gut (Erkosar Combe et al., 2014). More detailed analysis of the composition of excreta using spectrometric approaches may reveal the molecular consequences of these subtler intestinal effects.

Importantly, given the described lack of effects of the microbiota on the abovementioned parameters, we can conclude that the changes in pH and water balance observed upon dietary challenges and mating are attributable to physiological adaptations solely involving fly tissues. Hence, controlling for gut microbiota is not a major concern when designing faecal analysis experiments in *Drosophila*.

### 3.5 Infection status and its impact on intestinal physiology

The commensal nature of the relationship between the intestinal microbiota and their host may explain why intestinal readouts are not impacted by their presence. By contrast, non-commensal bacteria able to alter intestinal homeostasis and/or to trigger immune responses may affect intestinal physiology more severely, and their presence be reflected in alterations in excretion. To test this, we subjected flies to food-borne or systemic non-lethal bacterial infection and monitored their faecal patterns post-treatment.

**Fig. 6:**
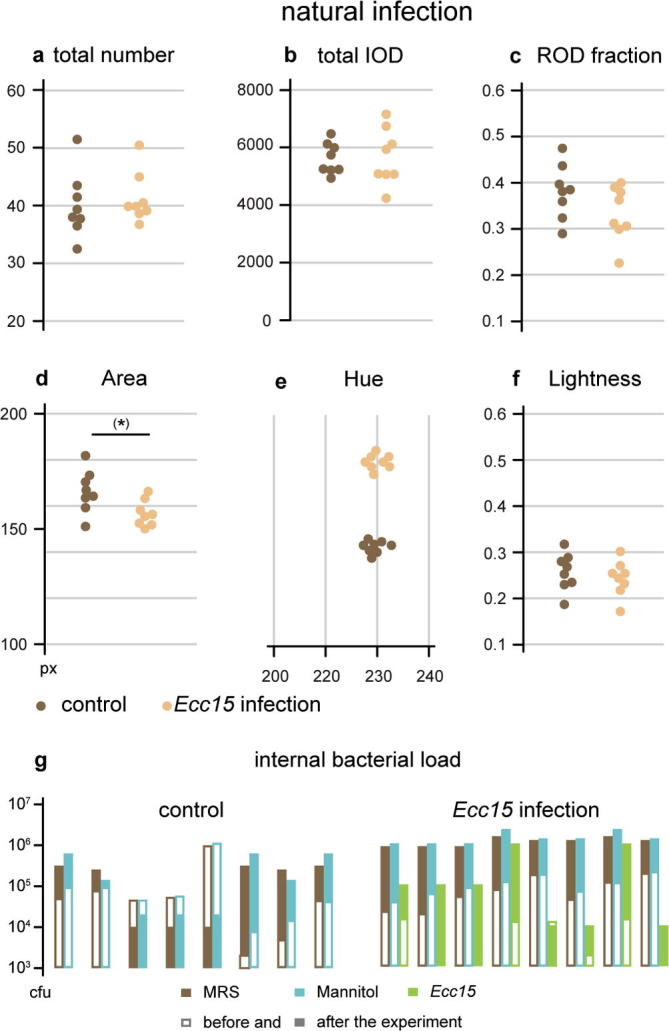
Faecal output patterns in food-borne infections. Natural infection with *E. carotovora carotovora*^15^ (yellow; control in brown) has no significant impact on various features of faecal output, including the number of spots (a), and the total excreted dye (b). RODs are present with normal frequency (c); spots have the same colour (acidity) and concentration as those produced by controls (e and f) but are slightly larger (d, significant only before repeated measure correction). Each of the replicates represents the faecal pattern of 8 adult female flies. The infection status of the flies was tested before and after the experiment (g, shown on a log scale) on MRS and Mannitol plates (for commensal bacteria) and on spectinomycin plates specific for the *Ecc15* bacterial strain. Only infected flies were tested for *Ecc15* as this strain is not naturally occurring and would not be contracted by untreated flies.

The non-pathogenic bacterium *E.* *carotovora carotovora*^15^ can infect *Drosophila* when ingested and results in a productive immune response coupled to efficient intestinal epithelium repair (Buchon et al., 2009b). Adult female flies subjected to 24 h treatment with Ecc15 suffer from gut infection associated with high bacterial loads (Fig. 6g) but their faecal output is largely normal when compared to uninfected controls (Fig. 6a–f), except for a small increase in deposit size of about 10% (Fig. 6d), which is significant only before correction. If confirmed, this effect might reflect physiological changes in intestinal passage associated with the infection, or simply a delayed overeating response due to aversion toward the infected food; in any case, the impact is limited when compared to the effects of mating or dietary manipulations.

**Fig. 7:**
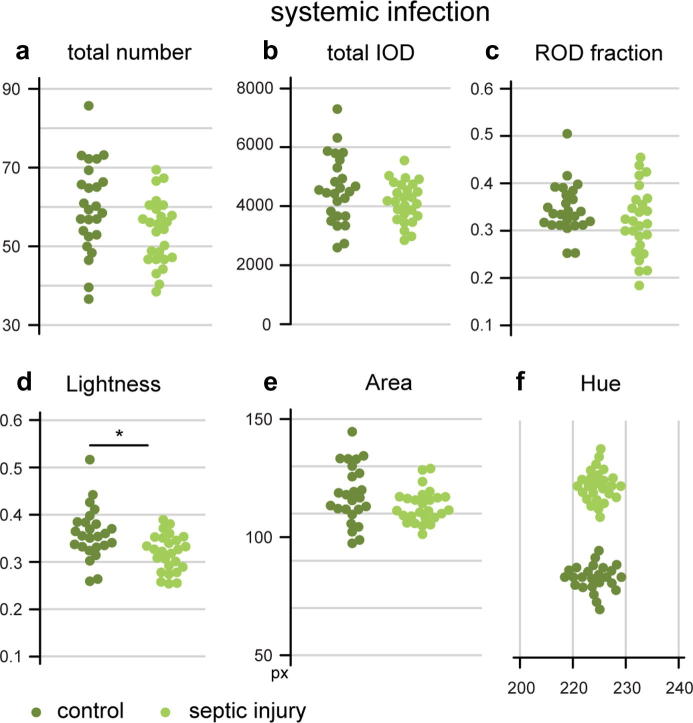
Faecal output patterns in systemic infections. A septic injury with a mixture of *M. luteus* and *E. coli* has no significant effect on the number (a) of spots, total excreted dye (b), and frequency of RODs (c), although both total number and IOD are slightly reduced. The Lightness value is significantly lower in septic injury samples (d), indicating that the excreta produced by infected flies are darker. Other graphical parameters such as size (e), colour (f) and shape (Circularity, not shown) are unaffected. Each of the replicates represents the faecal pattern of 8 adult female flies.

Infection by food-borne Ecc15 is contained within the gut and is eventually cleared completely (Basset et al., 2000). A wider engagement of the immune system can be triggered by a systemic infection, induced by septic injury (Lemaitre et al., 1997). However, the faecal output of flies subjected to septic injury was comparable to that of the uninjured control (Fig. 7a–f) in most aspects. A small (about 10%) but significant reduction in the Lightness variable indicates that the infection has an effect on water balance, whereby infected flies produce more concentrated excreta (Fig. 7d); however, the faecal spots also tended to be less abundant than in the controls (Fig. 7a), such that the total dye output was not higher, but in fact slightly lower (Fig. 7b). The small, non-significant reduction in the number of faecal spots and total dye excretion suggests that the overall amount of food processed by the gut may be reduced: an effect reminiscent of the metabolic disruption induced by certain persistent infections. In *Drosophila,* various pathogenic infections lead to anorexia (Ayres and Schneider, 2009) and a metabolic wasting effect occurs in flies infected with the pathogens *Mycobacter marinum* (Dionne et al., 2006) and *Listeria monocytogenes* (Chambers et al., 2012). In contrast with the treatments we tested, these infections are not cleared by the fly and result in death (Dionne et al., 2003, Mansfield et al., 2003). We therefore propose that such pathogenic bacteria could affect intestinal physiology more severely and may have a discernible effect on the faecal patterns, such as changes in appetite resulting in reduced overall output or alterations in the acid–base or water balance being reflected in the graphical features of excreta.

In sum, in the non-pathogenic situations that we have tested (Fig. 4, Fig. 5, Fig. 6, Fig. 7), we have found a remarkable consistency in the patterns and features of fly excreta upon alteration of their microbiota and infection status. The features highlighted by faecal spot analysis, such as acid-base balance and water reabsorption, are not heavily influenced by gut microbiota, food-borne gut infection or systemic infections delivered by septic injury; however, our experiments cannot rule out that other digestive processes might be. This possibility is supported by recent work reporting that the production of digestive enzymes (Erkosar Combe et al., 2014) and nutrient usage (Wong et al., 2014) can be regulated by the interplay of microbial and host metabolism. The changes associated with these alterations in microbiota status are not detected through faecal spot analysis and require more targeted or sophisticated approaches to be revealed. Nevertheless, changes in diet and mating status (Fig. 2, Fig. 4), as well as various previously reported genetic and neural manipulations (Cognigni et al., 2011) are readily identified by faecal spot analysis. Our results indicate that this assay can be reliably used to identify alterations in fly physiology and there is likely to be little to no confounding effect of gut microbiota and non-lethal infection in these conditions. We hope that the free software tool we have developed will support researchers interested in this assay and prove useful to the *Drosophila* community.
